# Comprehensive Diagnostic Nomogram for Predicting Clinically Relevant Postoperative Pancreatic Fistula After Pancreatoduodenectomy

**DOI:** 10.3389/fonc.2021.717087

**Published:** 2021-07-01

**Authors:** Bo Li, Ning Pu, Qiangda Chen, Yong Mei, Dansong Wang, Dayong Jin, Wenchuan Wu, Lei Zhang, Wenhui Lou

**Affiliations:** ^1^ Department of Pancreatic Surgery, Zhongshan Hospital, Fudan University, Shanghai, China; ^2^ Cancer Center, Zhongshan Hospital, Fudan University, Shanghai, China; ^3^ Department of General Surgery, Shangluo Central Hospital, Shaanxi, China

**Keywords:** pancreatic fistula, pancreaticoduodenectomy, risk factor, nomogram, decision curve analysis

## Abstract

**Background:**

Clinically relevant postoperative pancreatic fistula (CR-POPF) remains a severe and challenging complication of pancreaticoduodenectomy (PD). This study aimed to establish a novel postoperative nomogram-based diagnostic model for the early detection of CR-POPF in patients subjected to PD.

**Methods:**

Consecutive patients who underwent PD in Zhongshan Hospital, Fudan University from December 2018 to October 2020 were retrospectively enrolled. Univariate and multivariate logistic regression analyses were performed to identify independent risk factors for CR-POPF. Then, a novel predictive nomogram was established accordingly.

**Results:**

Among the consecutive 176 patients who underwent PD, 37 (21.1%) patients developed CR-POPF. Through univariate and multivariate analyses, the drain amylase (P = 0.002), serum creatinine (P = 0.009), and serum C reactive protein (P = 0.045) at postoperative day 1 (POD1) as well as the neutrophil count (P = 0.025) and temperature (P = 0.025) at POD3 were identified as independent risk factors for CR-POPF. Based on this, a novel predictive nomogram containing these factors was constructed to predict the probability of CR-POPF after PD. The formulated nomogram showed better performance to detect CR-POPF after PD with a sensitivity of 0.784, specificity of 0.770, positive predictive value of 0.475, and negative predictive value of 0.930 when compared to other predictors. In addition, the predictive value of the nomogram was assessed by a concordance index of 0.814 (95% CI, 0.736–0.892), which was significantly higher than indicators alone. This was further validated and depicted by decision curve analysis and clinical impact curve.

**Conclusion:**

This study established a diagnostic nomogram of postoperative objective parameters that can predict the development of CR-POPF after PD with a good discriminative ability and predictive accuracy.

## Introduction

Since Whipple reported three cases of pancreaticoduodenectomy (PD) for periampullary carcinoma in 1935 (1), PD has been the major surgical method for pancreatic head and periampullary malignancies. PD can also be performed to treat benign tumors and trauma of the pancreatic head and duodenum (2). Due to its complex surgical approach, long operation time and large trauma area, PD is considered as one of the most sophisticated and challenging surgeries within the gastrointestinal system (2, 3). With the development of perioperative management and the improvements in surgical techniques, the mortality of PD has declined (4). However, the mortality rate of postoperative complications remains at approximately 50% (5–7), of which postoperative pancreatic fistula (POPF) is the most common and severe postoperative complication (7–9).

As defined by the International Study Group of Pancreatic Surgeons (ISGPS) in 2016, POPF can be classified as biochemical leak (BL) and clinically relevant POPF (CR-POPF) (10). Nevertheless, only CR-POPF is regarded as a true pancreatic fistula, causing serious consequences such as postoperative bleeding, abdominal infection, and eventually death. Therefore, early prediction and timely intervention are necessary for the postoperative management of CR-POPF to promote the recovery of patients and reduce the cost of medical treatment and life-threatening events (9, 11). Previous studies found that there were several independent risk factors associated with CR-POPF, including age, main pancreatic duct (MPD) size, pancreatic texture, operative time, and operative blood loss (12–14). Predictive risk score models were established by incorporating these preoperative and intraoperative variables (9, 13, 15–17). However, since these predictors were pre- or intra-operative, they may be associated with anatomical characteristics of the pancreas, while postoperative indicators could provide clinical evidence for the early detection of CR-POPF. Nevertheless, recent studies showed that early postoperative blood factors, such as C-reactive protein (CRP), serum procalcitonin (PCT) and drain amylase (AMY), could be potential predictors of CR-POPF (18–21). The ideal early diagnostic model for CR-POPF should therefore incorporate postoperative factors, which has not been developed at present.

Therefore, this study aimed to identify postoperative risk factors of CR-POPF and establish a novel nomogram-based diagnostic model to predict CR-POPF after PD using a consecutive retrospective cohort of 176 patients. The predictive performances of this model were evaluated by the concordance index (C-index), calibration curve, decision curve analysis (DCA), and clinical impact curve (CIC).

## Methods

### Patients and Data Collection

In this study, consecutive patients with pancreatic or periampullary lesions who underwent PD in the Zhongshan Hospital, Fudan University from December 2018 to October 2020 were retrospectively included. All patients were enrolled according to the inclusion and exclusion criteria as follows (1): standard PD procedure performed (2); with complete preoperative examinations and postoperative 30-day follow-up data (3); without extended PD, combined with resections of other organs, palliative tumor resection, or vascular reconstruction (4); no preoperative infections or history of hematological and inflammatory diseases except for viral hepatitis. The protocol of this study was approved by the ethics committee of the Zhongshan Hospital, Fudan University.

General features of patients, preoperative radiological characteristics, operation details, and laboratory parameters were collected from the medical record. Among these, general features included age, gender, history of hypertension and diabetes, temperature (TEMP), and urinary output (UOP) on postoperative day 1 (POD1) and day 3 (POD3); the preoperative radiological characteristics included MPD diameter; the operation details included operation time and intraoperative bleeding; the laboratory parameters included red blood cells (RBC), hemoglobin (HGB), white blood cells (WBC), platelets (PLT), neutrophils (NEUT), lymphocytes (LYM), monocytes (MO), CRP, PCT, albumin (ALB), total bilirubin (TBil), alanine aminotransferase (ALT), aspartate aminotransferase (AST), γ-aminobutyric acid (γ-GT), alkaline phosphatase (ALP), Creatinine (Cr), blood urea nitrogen (BUN), operative area drainage (OAD), and drain AMY on POD1 and POD3. All data were collected by two independent surgeons and cross-checked.

### Operation

All patients underwent the standard Whipple procedure (22) performed by experienced attending surgeons who were able to independently perform more than 30 cases per year. The standard Whipple technique was performed as follows (1): first the Kocher technique was performed, the space between the descending part of the duodenum and the inferior vena cava was separated, the horizontal part of the duodenum from the retroperitoneum was freed, and the inferior vena cava, portal vein, and its branches were exposed (2); the gastrocolic ligament were then separated and the omentum sac entered to expose the tail of the pancreas (3); the anterior lobe of the transverse mesangial colon and dorsal pancreas were separated, and dissection was performed on the Henle trunk, right gastro-omental vein, superior anterior pancreaticoduodenal vein, accessory right colon vein, lower edge of the pancreatic head, and dorsal blood vessels (4); the hepatoduodenal ligament was dissected, and the blood vessels and bile ducts of the hilar exposed to free the common hepatic artery, right gastric artery, and splenic artery (5); a linear cutting closer was used to cut and retract the stomach, pylorus, and omentum, then the pancreas was exposed and its head and neck removed to free the uncinate process and superior mesenteric vein, the jejunum was also removed 20 cm away from the Treitz ligament (6); finally, pancreaticojejunostomy, cholangijejunostomy, and gastrojejunostomy were performed in this order.

### Postoperative Course and Follow-up

All patients received routine anti-infection, anticoagulation, and nutritional support after surgery, and no pancreatic enzyme inhibitors were used in the early stage. The parameters mentioned above on POD1 and POD3 were followed up.

According to the 2016 edition of the ISGPF, PF was diagnosed and graded (10), emphasizing the correlation between the grade of PF and clinical outcome. It is believed that the diagnosis of PF can only be made when the clinical course changes. According to the severity of the clinical outcome, the previous grade A PF was renamed as BL in which no intervention is necessary. Grade B PF requires invasive operations including interventional angiography and puncture drainage, while grade C PF refers to combined organ dysfunction, secondary operation, or death. CR-POPF contains only grades B and C PF.

### Definitions

PD is one of the most important operations in general surgery. Due to the large scope of surgery involved, removal of many organs, and complex reconstruction of digestive tract, postoperative complications are frequent and complex. Among them, PF is quite common with a reported incidence of more than 30% (15, 23). It mainly includes: BL (grade A PF) related to pancreatic parenchymal leakage; and pancreaticojejunostomy-related anastomotic fistula (grades B and C PF) (10). The pathogenesis, treatment measures, and prognosis of these two are quite different, and even complicated by severe abdominal infection, bleeding, and even death. However, the current definition and grading of PF, especially CR-POPF that have a greater impact on the patient’s recovery, can only be accurately diagnosed after a long period of surveillance, which is just dependent on time (10, 24). Early diagnosis of CR-POPF after surgery remains a vital issue.

### Statistical Analysis

In this study, the SPSS 26.0 software (SPSS Inc., Chicago, IL, USA) and R 4.0.2 software (http://www.r-project.org/) were used for data analysis. Categorical variables were presented as frequencies and percentages, and compared by Chi-square test or Fisher’s exact test. Continuous variables were displayed as the median and interquartile range (IQR), and compared by Student’s t-test or Mann–Whitney U test, as appropriate. Independent risk factors were analyzed by univariate and multivariate logistic regressions. The optimal cut-off values, sensitivity, specificity, positive predictive value (PPV), and negative predictive value (NPV) were determined by receiver operating characteristic curve (ROC) analysis. P < 0.05 indicated statistical significance.

The diagnostic nomogram was constructed with R project software to predict CR-POPF, and its performance was measured by C-index, calibration curve, DCA, and CICs (25, 26).

## Results

### Demographic and Clinicopathological Characteristics

Based on the inclusion and exclusion criteria, a total of 176 patients were enrolled in this study. The median age of the patients was 64.5 years (IQR: 56.0–71.0). In this cohort, there were 98 males (55.7%) and 78 females (44.3%), 58 (33.0%) patients with hypertension and 44 (25.0%) patients with diabetes. Before surgery, the Ca19-9 was detected and its median value was 24.3 U/ml (IQR: 10.0–110.8), and MPD dilation was observed in 119 (67.6%) patients. Moreover, the median operative time was 4.0 h (IQR: 3.5–4.5) and the median volume of bleeding was 100 ml (IQR: 100–200). After surgical operation, the median tumor size was 2.3 cm (IQR: 1.8–3.0), and most of the tumors (124, 70.5%) were confirmed as malignancies.

During the postoperative follow-up and management, there were 139 (78.9%) patients who did not present CR-POPF, while 37 (21.1%) patients experienced CR-POPF. Parameters collected on POD1 and POD3 are shown in **Table 1**.

**Table 1:** Demographic and clinical characteristics of patients with CR-POPF following PD.

Variables	No CR-POPF (n = 139)	CR-POPF (n = 37)	P value
**General parameters**			
Male, n (%)	75 (54.0)	23 (62.2)	0.372
Age (years), median (IQR)	64.0 (55.0, 71.0)	67.0 (58.5, 71.5)	0.378
Hypertension, n (%)	44 (31.7)	14 (37.8)	0.477
Diabetes, n (%)	34 (24.5)	10 (27.0)	0.749
MPD dilation, n (%)	97 (69.8)	22 (59.5)	0.233
Ca19-9 ≥37, n (%)	59 (42.4)	19 (51.4)	0.333
Operation time >4 h, n (%)	51 (36.7)	6 (16.2)	**0.018**
Bleeding ≥400 ml, n (%)	13 (9.4)	6 (16.2)	0.369
Malignant tumor, n (%)	99 (71.2)	25 (67.6)	0.665
Tumor size, median (IQR)	2.50 (1.80, 3.00)	2.20 (1.75, 3.00)	0.758
Lymph node metastasis, n (%)	28 (20.1)	7 (18.9)	0.868
Vascular invasion, n (%)	49 (35.3)	16 (43.2)	0.371
Neural invasion, n (%)	67 (48.2)	18 (48.6)	0.961
**POD1 parameters**			
TEMP, median (IQR)	37.5 (37.1, 37.8)	37.5 (37.2, 37.8)	0.353
UOP, median (IQR)	1,650 (1,300, 1,970)	1,400 (1,100, 1,750)	**0.018**
TBil, median (IQR)	24.0 (14.3, 74.2)	27.0 (17.4, 40.3)	0.683
ALB, median (IQR)	35.0 (32.0, 38.0)	35.0 (32.0, 37.5)	0.755
ALT, median (IQR)	54.0 (27.0, 100.0)	47.0 (32.0, 77.0)	0.880
AST, median (IQR)	47.0 (33.0, 80.0)	55.0 (36.5, 73.0)	0.794
γ-GT, median (IQR)	77.0 (17.0, 274.0)	51.0 (24.5, 135.0)	0.575
ALP, median (IQR)	92.0 (57.0, 190.0)	67.0 (53.5, 133.5)	0.131
RBC*10^12^, median (IQR)	4.03 (3.53, 4.38)	4.07 (3.62, 4.40)	0.575
HGB, median (IQR)	122.0 (109.0, 134.0)	124.0 (113.5, 137.0)	0.328
PLT*10^9^, median (IQR)	200.0 (154.0, 244.0)	215.0 (180.5, 227.0)	0.529
WBC*10^9^, median (IQR)	11.98 (9.73, 14.45)	13.21 (11.18, 14.69)	0.093
NEUT*10^9^, median (IQR)	10.2 (8.3, 12.6)	11.4 (9.2, 12.8)	0.133
LYM*10^9^, median (IQR)	0.8 (0.6, 1.2)	0.8 (0.6, 1.1)	0.780
MO*10^9^, median (IQR)	0.71 (0.53, 0.91)	0.86 (0.74, 1.07)	**0.001**
CRP, median (IQR)	35.5 (21.8, 54.5)	45.1 (27.6, 64.6)	**0.018**
PCT, median (IQR)	0.26 (0.18, 0.49)	0.33 (0.25, 0.62)	**0.034**
Cr, median (IQR)	69.0 (60.0, 84.0)	84.0 (63.5, 99.5)	**0.017**
BUN, median (IQR)	4.7 (3.3, 5.7)	4.9 (4.0, 6.4)	0.147
OAD, median (IQR)	150.0 (84.0, 240.0)	180.0 (110.0, 300.0)	0.154
AMY*10^3^, median (IQR)	1105.0 (183.0, 4,487.0)	5,650.0 (2,273.5, 1,1738.0)	**<0.001**
**POD3 parameters**			
TEMP, median (IQR)	37.0 (36.8, 37.3)	37.3 (36.9, 37.6)	**0.006**
UOP, median (IQR)	1,800 (1,400, 2,100)	1,600 (1,240, 1,975)	0.097
TBil, median (IQR)	22.6 (14.1, 49.7)	22.9 (15.8, 62.4)	0.478
ALB, median (IQR)	36.0 (34.0, 38.0)	35.0 (32.5, 37.0)	0.152
ALT, median (IQR)	31.0 (16.0, 56.0)	28.0 (16.5, 45.5)	0.660
AST, median (IQR)	26.0 (21.0, 41.0)	26.0 (18.5, 35.5)	0.373
γ-GT, median (IQR)	48.0 (16.0, 170.0)	46.0 (15.0, 79.0)	0.348
ALP, median (IQR)	79.0 (55.0, 149.0)	66.0 (51.5, 99.5)	0.053
RBC*10^12^, median (IQR)	3.37 (3.05, 3.75)	3.47 (3.06, 3.73)	0.946
HGB, median (IQR)	104.0 (95.0, 114.0)	106.0 (93.0, 115.5)	0.734
PLT*10^9^, median (IQR)	166.0 (128.0, 210.0)	148.0 (132.0, 203.5)	0.638
WBC*10^9^, median (IQR)	8.61 (6.64, 11.09)	11.16 (8.36, 13.24)	**0.007**
NEUT*10^9^, median (IQR)	7.1 (5.2, 9.3)	9.2 (7.5, 11.7)	**<0.001**
LYM*10^9^, median (IQR)	1.0 (0.7, 1.3)	0.9 (0.7, 1.3)	0.783
MO*10^9^, median (IQR)	0.62 (0.48, 0.81)	0.74 (0.63, 1.00)	**0.006**
CRP, median (IQR)	91.3 (46.5, 149.1)	114.3 (67.4, 208.4)	**0.025**
PCT, median (IQR)	0.20 (0.12, 0.38)	0.3 (0.18, 0.65)	**0.008**
Cr, median (IQR)	64.0 (52.0, 76.0)	70.0 (53.5, 88.5)	0.055
BUN, median (IQR)	4.5 (3.1, 5.3)	4.6 (3.6, 6.0)	0.156
OAD, median (IQR)	151.0 (64.0, 350.0)	245.0 (80.0, 452.5)	0.132
AMY*10^3^, median (IQR)	933.0 (88.0, 2,984.0)	1,954.0 (597.0, 6,059.0)	**0.008**

OAD, operative area drainage. The bold values mean the P value < 0.05.

### Correlations Between CR-POPF and Clinical Parameters

Demographic and clinical characteristics of patients with or without CR-POPF are displayed in **Table 1**. There was no significant relationship observed between CR-POPF and gender, age, concomitant diseases (hypertension and diabetes), bleeding, MPD dilation, Ca19-9, tumor size, lymph node metastasis, vascular invasion, or neural invasion. Among POD1 parameters, UOP (P = 0.018), MO (P = 0.001), CRP (P = 0.018), PCT (P = 0.034), Cr (P = 0.017), and drain AMY (P < 0.001) were significantly correlated with CR-POPF, while for POD3 parameters, NEUT (P = 0.006), WBC (P = 0.007), NEUT (P < 0.001), MO (P = 0.006), CRP (P = 0.025), PCT (P = 0.008), and AMY (P = 0.008) were positively correlated with CR-POPF.

### Independent Risk Factors Associated With CR-POPF

**Figure 1:**
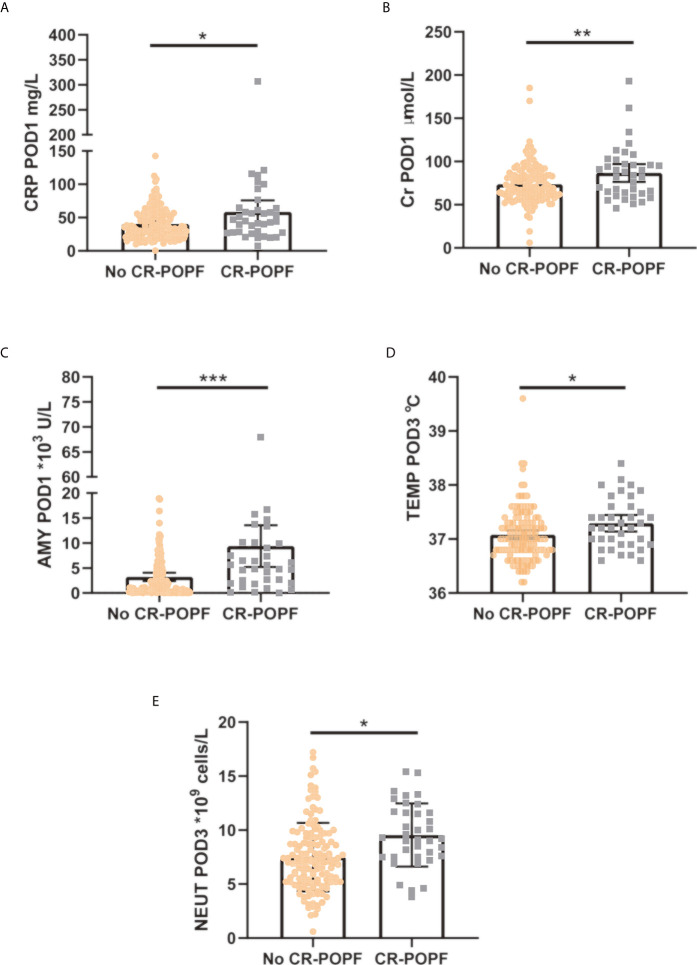
Quantification of CRP at POD1 **(A)**, Cr at POD1 **(B)**, AMY at POD1 **(C)**, TEMP at POD3 **(D)**, and NEUT at POD3 **(E)** distinguishing patients with CR-POPF from those without CR-POPF. The error bars represent median ± standard deviation. CRP at POD1, C-reactive protein at postoperative day 1; Cr at POD1, Creatinine at postoperative day 1; AMY at POD1, amylase of drainage at postoperative day 1; TEMP at POD3, temperature at postoperative day 3; NEUT at POD3, neutrophils at postoperative day 3; CR-POPF, clinically relevant postoperative pancreatic fistula. *P < 0.05, **P < 0.01, ***P < 0.001.

To identify independent risk factors associated with CR-POPF, a univariate logistic regression analysis was performed and showed that operation time (P = 0.022), MO at POD1 (P = 0.022), CRP at POD1 (P = 0.010), PCT at POD1 (P = 0.036), Cr at POD1 (P = 0.009), drain AMY at POD1 (P < 0.001), TEMP at POD3 (P = 0.015), WBC at POD3 (P = 0.026), NEUT at POD3 (P = 0.001), MO at POD3 (P = 0.012), CRP at POD3 (P = 0.004), and drain AMY at POD3 (P = 0.008) were significant risk factors for CR-POPF. In addition, the multivariate analysis showed that CRP at POD1 (P = 0.045, OR = 1.013, 95% CI: 1.000–1.026), Cr at POD1 (P = 0.028, OR = 1.019, 95% CI: 1.002–1.036), drain AMY at POD1 (P = 0.002, OR = 1.111, 95% CI: 1.041–1.185), TEMP at POD3 (P = 0.025, OR = 2.714, 95% CI: 1.130–6.516), and NEUT at POD3 (P = 0.025, OR = 1.171, 95% CI: 1.020–1.344) were considered independent risk factors for CR-POPF (**Table 2**). Indeed, levels of CRP at POD1 (**Figure 1A**), Cr at POD1 (**Figure 1B**), drain AMY at POD1 (**Figure 1C**), TEMP at POD3 (**Figure 1D**), and NEUT at POD3 (**Figure 1E**) were significantly higher in CR-POPF patients when compared to those without CR-POPF, which may be of great value in early prediction of CR-POPF after PD.

**Table 2:** Univariate and multivariate logistic regression analysis for CR-POPF in patients following PD.

Variables	Univariable P value	Multivariate P value	β	OR	95% CI
**Operation time**	**0.022**	0.090*	−0.949	0.384	0.129–1.161
**UOP POD1**	0.775	**-**	–	–	–
MO*10^9^ POD1	**0.022**	0.777*	0.240	1.271	0.242–6.688
**CRP POD1**	**0.010**	**0.045**	0.013	1.013	1.000–1.026
**PCT POD1**	**0.036**	0.179*	0.505	1.658	0.793–3.465
**Cr POD1**	**0.009**	**0.028**	0.019	1.019	1.002–1.036
AMY*10^3^ POD1	**<0.001**	**0.002**	0.105	1.111	1.041–1.185
**TEMP POD3**	**0.015**	**0.025**	0.998	2.714	1.130–6.516
WBC*10^9^ POD3	**0.026**	0.651*	0.005	1.005	0.983–1.028
NEUT*10^9^ POD3	**0.001**	**0.025**	0.158	1.171	1.020–1.344
MO*10^9^ POD3	**0.012**	0.363*	0.818	2.265	0.389–13.195
**CRP POD3**	**0.004**	0.452*	−0.003	0.997	0.990–1.004
**PCT POD3**	0.127	–	–	–	–
AMY*10^3^ POD3	**0.008**	0.252*	0.055	1.056	0.962–1.160

β, regression coefficient; OR, odds ratio; CI, confidence interval; *In the multivariate logistic regression analysis, variables (p > 0.05) were excluded from the final model based on the results of the backwards stepwise analysis. The bold values mean the P value < 0.05.

### Construction of a Predictive Nomogram Incorporating Risk Factors for CR-POPF

**Figure 2:**
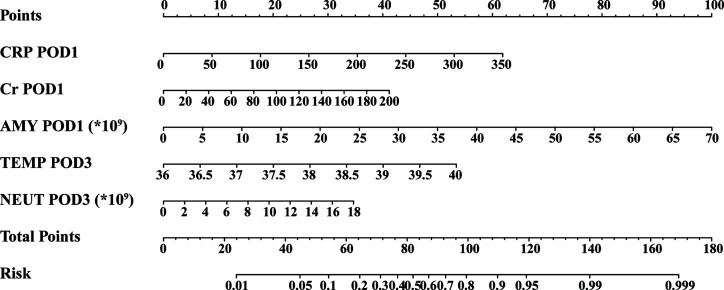
Predictive nomogram for the probability of clinically relevant postoperative pancreatic fistula. *, a multiplication sign.

**Figure 3:**
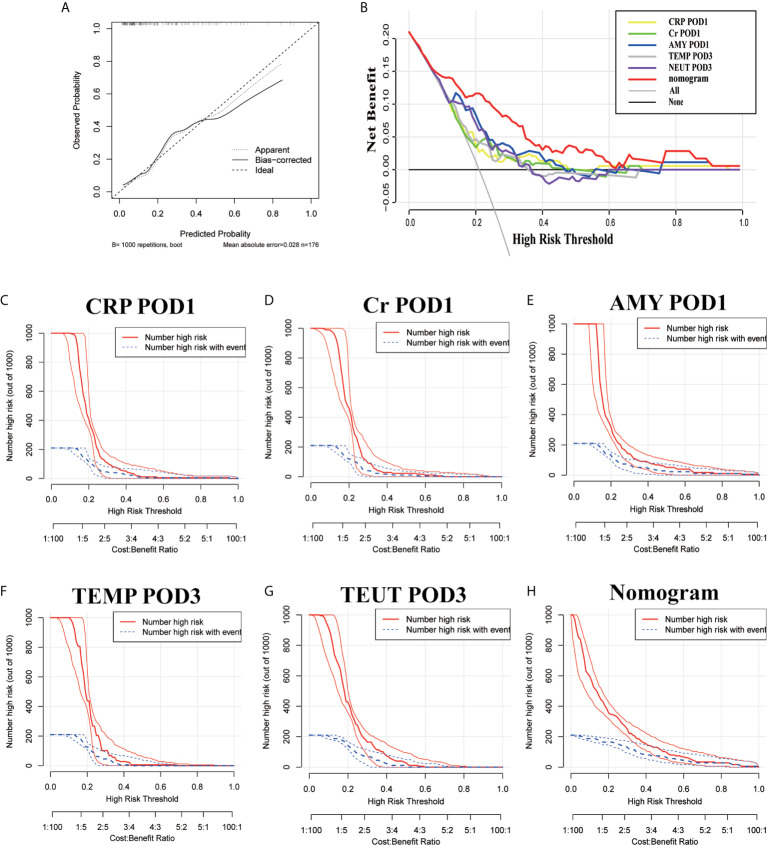
The calibration curves **(A)**, decision curve analysis **(B)**, and a clinical impact curve **(C–H)** of the nomogram and risk factors.

Based on the above results and to create an accurate predictive model, we integrated five independent predictive indicators, namely CRP, Cr, drain AMY at POD1, and TEMP, NEUT at POD3 to construct a novel predictive nomogram (**Figure 2**). The C-index of the formulated nomogram was 0.814 (95% CI, 0.736–0.892), which was significantly higher than that of each indicator alone [CRP at POD1: 0.627 (95% CI, 0.524–0.729); Cr at POD1: 0.627 (95% CI, 0.519–0.736); drain AMY at POD1: 0.746 (95% CI, 0.658–0.834); TEMP at POD3: 0.637 (95% CI, 0.538–0.737); NEUT at POD3: 0.698 (95% CI, 0.607–0.790)]. The formulated nomogram showed better discriminatory performance to detect CR-POPF with a sensitivity of 0.784, specificity of 0.770, PPV of 0.475, and NPV of 0.930 when compared to other models (**Table 3**). The calibration curves exhibited optimal consistency between actual observations and nomogram-predicted CR-POPF (**Figure 3A**).

**Table 3:** Discriminatory performance of CRP, Cr, AMY, TEMP, NEUT, and the formulated nomogram for detecting patients with CR-POPF after PD.

Variables	AUC (95% CI)	Cut-off level	Sensitivity	Specificity	PPV	NPV
**CRP POD1**	0.627 (0.524–0.729)	39.45	0.649	0.604	0.304	0.866
**Cr POD1**	0.627 (0.519–0.736)	87.5	0.486	0.777	0.367	0.850
**AMY POD1**	0.746 (0.658–0.834)	2.52	0.757	0.662	0.373	0.911
**TEMP POD3**	0.637 (0.538–0.737)	37.35	0.432	0.777	0.340	0.837
**NEUT POD3**	0.698 (0.607–0.790)	7.45	0.811	0.576	0.337	0.920
**Nomogram**	0.814 (0.736–0.892)	65.50	0.784	0.770	0.475	0.930

AUC, area under the receiver-operating-characteristic curve; CI, confidence interval; PPV, positive predictive value; NPV, negative predictive value.

The predictive accuracy of this nomogram was further confirmed by DCA and CIC, which are novel evaluation methods to highlight prediction models with clinical net benefit. Indeed, the constructed nomogram showed superior net benefit with a wider range of high-risk thresholds when compared to each indicator alone, which meant improved performance for the prediction of CR-POPF (**Figure 3B**). Finally, the nomogram with higher risk threshold probability levels and a smaller gap between actual and predicted curves represented superior estimation of decision outcomes (**Figures 3C–H**).

## Discussion

CR-POPF remains one of the most common and severe complications of PD. It can increase the length of hospitalization and economic burden of patients, while causing lethal outcomes, such as abdominal infection and bleeding (27, 28). Abdominal drainage is routinely used to prepare for postoperative complications after PD. However, the routine long-term drainage for patients with a low risk of developing CR-POPF might increase the probability of intra-abdominal infections (29, 30). The early identification of low-risk patients can improve the postoperative management of patients subjected to PD.

Although several risk factors have been associated with CR-POPF, a single factor could not accurately predict it. Therefore, various models integrating multiple independent risk factors have been established to predict CR-POPF after PD. However, many studies have constructed models with only preoperative and intraoperative factors, ignoring postoperative factors (9, 13, 15–17). It has been reported that the early removal of drains at POD4 could decrease the occurrence of intra-abdominal infections after PD (29). Thus, biochemical parameters at POD1 and POD3 were collected and used to predict the risk of developing CR-POPF in this study. A nomogram-based diagnostic model was constructed by combining CRP, Cr and drain AMY at POD1, and TEMP, NEUT at POD3. This diagnostic model has showed good discriminative ability and predictive accuracy for CR-POPF.

It has been recognized that rises in CRP, TEMP, and NEUT are clinical signs of inflammation, while leakage of enzyme-rich pancreatic juice into the abdomen may induce inflammation (31). Unsurprisingly, these factors could be suitable for the early detection of CR-POPF. Indeed, many studies have shown that increased CRP (32–34) and leakage after colorectal surgery (35) are strongly associated with CR-POPF after PD. Meanwhile, levels of AMY on the abdominal drain at POD1 have been widely used to predict CR-POPF with high accuracy (36–38). However, some researchers have contested this claim (39). On one hand, AMY might not be elevated when abdominal abscesses develop due to latent POPF (40). On the other hand, many patients with BL may also have a relatively high AMY at POD1 (41). Thus, it is necessary to combine several factors to reliably predict CR-POPF. Furthermore, our result indicated that patients with a high level of Cr at POD1 tended to have a higher risk of CR-POPF, which has not been described before. Previous studies have reported that preoperative Cr or asymptomatic renal dysfunction led to a higher risk of CR-POPF after PD (42, 43). Finally, we found that PCT levels at POD1 were a significant risk factor for CR-POPF only in our univariate analysis. PCT showed poorer predictive performance than CRP and drain AMY, which was consistent with previous studies (21, 44).

Currently, several studies have attempted to develop predictive models for CR-POPF after PD using soft pancreatic parenchyma as a parameter, which might be a challenge for reconstruction and might be associated with increased exocrine activity (9, 45–47). However, there is no accurate criterion to define soft pancreatic parenchyma. The judgment of “pancreatic texture” is drawn by the surgeon’s subjective touch. Different people or measurement methods will produce large deviations, so we did not assess this intraoperative parameter. In our study, no preoperative or intraoperative factors were significant in the multivariate analysis, which differs from previous studies. The reason might be that several postoperative biochemical indicators were also included in the analysis, suggesting that these postoperative clinical and laboratory parameters showed higher sensitivity and specificity than preoperative or subjective intraoperative indicators.

Compared with previous predictive models, our nomogram-based diagnostic model for CR-POPF is superior. Kawai et al. (29) found that drain removal at POD4 could reduce the incidence of intra-abdominal infection after PD. It is known that early removal of the drainage tube can be beneficial to the recovery of patients without CR-POPF. However, early drain removal should be performed with extreme caution. It would be harmful to those with CR-POPF since the leak of pancreatic juice without drain could contribute to intra-abdominal infections and bleeding. Our novel predictive model could accurately stratify patients with different risks of CR-POPF based on well-known clinical and laboratory parameters performed at POD1 and POD3, suggesting that our model can help surgeons make personalized postoperative management to prevent and minimize complications after PD. For patients with high risk of CR-POPF, we can apply the somatostatin, antibiotics, and parenteral nutrition at the early phase, prolong the fasting time and gastrointestinal drainage, and examine abdominal enhanced CT, drainage culture, etc. to prevent aggravation of CR-POPF and the development of secondary infection. Moreover, all postoperative parameters incorporated in this nomogram-based diagnostic model were objective and easily obtained. Therefore, this model is promising for clinical application.

However, there were still certain limitations to our study. First, this is a single-center retrospective study with limited sample size and the PPV was also relatively low, so the cut-off values for variables may not be suitable for other researches, and large-cohort, multicenter, prospective studies or relevant meta-analyses are required to determine the optimal cut-off values, and risk thresholds for stratifying patients should be determined in further studies. Second, our nomogram based on postoperative findings could not be applied to prevent CR-POPF before surgery. Thus, it would be better to be combined with other models based on the preoperative parameters. If patients were identified as the high risk of CR-POPF before surgery, chemical pancreatic duct occlusion to avoid pancreatic anastomosis might be a good choice as previous reported (48). If patients were identified as the low risk of CR-POPF according the preoperative models, surgical procedure was performed and then our nomogram based on postoperative indicators, which showed a higher sensitivity and specificity, could be applied to monitor aggravation of CR-POPF.

In conclusion, postoperative clinical and laboratory parameters such as CRP, Cr and drain AMY at POD1, and TEMP and NEUT at POD3 are independent predictive factors of CR-POPF. The novel nomogram-based diagnostic model was established based on the above parameters and shows good discriminative ability and predictive accuracy. This is a promising tool to predict CR-POPF after PD, to improve personalized management and improve the recovery of PD patients.

## Data Availability Statement

The raw data supporting the conclusions of this article will be made available by the authors, without undue reservation.

## Ethics Statement

The studies involving human participants were reviewed and approved by the Ethics Committee of Zhongshan Hospital, Fudan University. The patients/participants provided their written informed consent to participate in this study.

## Author Contributions

Study concept and design: BL, NP, and WL. Acquisition of data: BL and LZ. Statistical analysis: NP. Drafting of manuscript: BL, NP, and QC. Critical revision of manuscript for important intellectual content: BL, NP, QC, YM, DW, DJ, WW, LZ, and WL. All authors contributed to the article and approved the submitted version.

## Funding

This work is funded by the Shanghai Sailing Program (21YF1407100), China Postdoctoral Science Foundation (2021M690037), National Key R&D Program (2019YFC1315902), and Clinical Science and Technology Innovation Project of the Shanghai ShenKang Hospital Development Centre (SHDC2020CR2017B).

## Conflict of Interest

The authors declare that the research was conducted in the absence of any commercial or financial relationships that could be construed as a potential conflict of interest.
